# Nexus between Air Pollution and Neonatal Deaths: A Case of Asian Countries

**DOI:** 10.3390/ijerph16214148

**Published:** 2019-10-28

**Authors:** Asim Anwar, Muhammad Ayub, Noman Khan, Antoine Flahault

**Affiliations:** 1 Department of Management Sciences, COMSATS University Islamabad, Attock Campus, Punjab 43600, Pakistan; m.ayub@ciit-attock.edu.pk (M.A.); nomankhan1010@hotmail.com (N.K.); 2 Swiss School of Public Health (SSPH+), Hirschengraben 82, 8001 Zürich, Switzerland; antoine.flahault@unige.ch; 3 Institute of Global Health, Faculty of Medicine, University of Geneva, CH-1202, 8001 Geneva, Switzerland

**Keywords:** air pollution, temperature, acute respiratory infection, neonates’ deaths, Asian countries

## Abstract

The rapid economic growth in Asian countries has witnessed a persistent increase in air pollution complementing adverse health challenges for children in these countries. Quantification of health effects attributable to air pollution (PM_2.5_) is important in policy implications to tackle air pollution and associated health problems. This study aims to explore the nexus between air pollution and neonates’ deaths embedded in acute respiratory infection. We collected panel data from the 12 most vulnerable Asian countries over the period of 2000–2017 and analyzed through the fixed-effect model. Empirical results show a positive relation between air pollution, temperature, and neonates’ deaths in the studied Asian countries. The results have attested negative impacts of income and education while positive effect of population density on neonates’ deaths due to acute respiratory infection. Diagnostic and prognostic measures have checked the pace of the respiratory diseases caused by PM_2.5_ and resultant deaths in Asian countries; yet alarming factors, like mounting industrial air pollution and rapid expansion of industrial zones in urban areas, need to be addressed in policy implications for long term sustainable solutions.

## 1. Introduction

Air pollution has become the worst environmental health-related challenge in the world. The World Health Organization (WHO) has reported that 22% of global deaths and disabilities are elicited by environmental factors [1]. Unfortunately, 92% of the world’s population has been estimated to breathe unhealthy polluted air that is exceeding health-based limits [2]. Fossil fuel consumption for higher economic growth causes evasive air pollution through emission of pollutants like sulfur dioxide (SO_2_), nitrogen dioxide (NO_2_), ozone (O_3_), and particulate matter (PM) [3]. WHO has asserted air pollution as the worst recent threat being faced by global public health, which is bigger in growth rate as compared to HIV or Ebola [4].

The global burden of disease has underlined air pollution as the major stimulus for global disease burden specifically with reference to the developing countries [5,6]. Most of the developing countries push to consume low-cost energy sources for domestic and industrial purposes but inadvertently contribute to air pollution due to dearth of mitigating technologies [7]. The ambient air pollution is estimated to have caused 4.2 million deaths in 2016 where 18% were attributed to acute lower respiratory infections [8]. Air pollution appears in the form of substantial pollutants variant in size and chemical nature, either accumulating or going through the lungs’ tissues by distressing the respiratory tract mitigating system, which is made up of different mucosal cilia and protective mechanisms [9]. Children face more susceptibility when exposed to air pollution and subsequent increased risk of acute respiratory infections, for diverse reasons [10,11]. Firstly, the epithelial linings of children’s lungs are not so well developed and show strong permeability for pollutants [12]; secondly, children’s immune system is in the developing stage, and has limited body defense to counter infection [13]; and thirdly children experience higher respiration rates due to relatively larger lung surface area with proportion to their body weights as compared to adults [14]. Usually, vapors of pollutants are either absorbed through human tissues or processed as body fluids depending on hydrophilicity and hydrophobicity, but air pollutants like PM_2.5_ enter the lungs and are a notable health risk determinant [15,16,17]. Ambient air pollution (PM_2.5_) severely victimizes people having a history of respiratory disease like chronic obstructive pulmonary disease (COPD) [5,18]. The literature has validated the contributive impact of air pollution (PM_2.5_) in onset of various respiratory diseases like respiratory infection, chronic obstructive pulmonary disease (COPD), asthma, and lung cancer [19,20,21,22,23]. [24] found strong association between air pollution and respiratory disease mortalities and outpatient visits in southeastern China. [25] verified the positive association between air pollution and upper respiratory and lower respiratory infections and consequent deaths in infants and children in four large Latin-American cities. [26] also testified to a strong association between carbonaceous PM_2.5_ and infant mortality, especially neonatal mortality in 43 low and middle income countries. Acute Respiratory Infection (ARI) was labeled as the sixth leading death factor for all ages, and specifically, the major cause of death in children less than 5 years age [11].

Mortality of children under 5 years has been significantly controlled and redressed in the last two decades, ranging from 91 deaths in 1990 to 43 deaths per 1000 live births in 2015 [13]. But, still the mortality of neonates aging less than 28 days’ life span and infants aging less than a year is higher with the ratio of 2/3 of under 5 deaths in children less than 5 years of age, and most alarmingly, 90% of these deaths took place in less developed countries [14,27]. The WHO and maternal and child epidemiology (MCEE) estimates have reported 159,416 worldwide neonatal deaths attributed to acute respiratory infections in 2016 having a declining trend as shown in Table 1. 

In literature, various studies have been conducted with reference to to air pollution and mortality in children caused by acute respiratory infection but scarce attention has been paid to address the neonates’ mortality complemented by air pollution. Therefore, the present study, firstly, contributes in the literature regarding air pollution and its pernicious impacts on neonatal mortality caused by acute respiratory infection in Asian countries, which have been experiencing an ever increasing shift in air pollution (PM_2.5_) since 2010. Secondly, the previous studies assessing the relationship between air pollution and respiratory diseases either have mostly focused on single country or different cities within a country, while the present study employs the panel dataset to check the impact of air pollution on neonatal deaths due to acute respiratory infection in a panel of 12 Asian countries including Bangladesh, Sri Lanka, India, Pakistan, Mongolia, Myanmar, China, Nepal, Indonesia, Philippine, Vietnam, and Thailand. 

## 2. Data and Methodology

### Empirical Model

The study has employed a similar empirical model [28] as the one that studied the association between climate change and infectious diseases. The present study has extended the framework by associating climate change, air pollution (PM_2.5_), and socio-economic variables causing neonates deaths due to acute respiratory infection. This study has used panel data of 12 Asian countries from 2000 to 2017. The empirical model is mentioned below:

(1) M=g(CV,D),

where *M* denotes the number of neonates deaths due to acute respiratory infection, *CV* signifies the climate variables (application of mean temperature as a proxy for *CV*) and air pollution (PM_2.5_), while *D* signifies socio-economic variables, i.e., income, education, and population density. The socio-economic determinants have been added in the model to check the rationality of the results. We describe the climate variables (air pollution and temperature) as non-linear. The objective behind introducing the climate variables in quadratic form is to determine the number of neonatal deaths caused by respiratory disease patients as non-linear in climate variables or otherwise. The study has estimated a static model where the current number of neonates’ deaths is not dependent upon the previous year number of deaths. The motive behind using the static model is that the data available are annual. Thus, the static model is expressed as:

(2) $$M_{it}=\beta_{0}+\beta_{1}temp_{it}+\beta_{2}PM_{2.5_{it}}+\delta D_{it}+\varepsilon_{it}$$

The study employs the panel dataset method to evaluate the effects of air pollution (PM_2.5_) and climate change (temperature) on the neonate deaths due to acute respiratory infection. It is important to state here that the present data structure does not permit to analyze the random effects. According to [29], the fixed-effect model is a suitable specification if the analysis is focused on a given number (N) of units so that statistical inference is conditional on the particular set of (N) unities, which in our case, are 12 countries highly vulnerable to climate change (N = 12). On the other hand, random-effect models require the assumption of uncorrelated explanatory variables and a time-invariant unobservable component of the model, which is assumed to be random (for example, [30,31]). In other words, the random effect model would require that units were selected randomly from a large number of possibilities, as is the case when the units are individuals or households. The static model is elaborated below as:

(3) $$\begin{array}{cc} Neonatal\;deaths_{it} & \\ & =\beta_{0}+\beta_{1}PM_{2.5_{it}}+\beta_{2}PM_{2.5_{it}}^{2}+\beta_{3}temp_{it}+\beta_{4}temp_{it}^{2}\\ & +\beta_{5}GDP_{it}+\beta_{6}Edu_{it}+\beta_{7}PopDen_{it}+\varepsilon_{it} \end{array}$$

The study uses a panel dataset where each variable in Equation (3) refers to country *i* at time *t*, while *D_it_* in Equation (2) denotes socio-economic factors (Gross Domestic Product (GDP) as a measure of income, education level, and population density), PM_2.5_ refers to air pollution (mean annual exposure (micrograms per cubic meter)), *temp_it_* is used as a proxy for climate change, and ε*_it_* is the error term. The study measures the neonatal deaths due to acute respiratory infections per 1000 inhabitants as the dependent variable with reference to retrieved data from United Nation International Emergency Fund (UNICEF) database. The data for other variables, like temperature (yearly average of monthly mean temperature of respective countries), GDP per capita (as a proxy for national income), secondary school enrollment (as a proxy for national educational level), and population density (people per sq. km of land area) are retrieved from the World Bank dataset.

## 3. Results

We have transformed all the variables into logarithmic form except temperature and applied Levin–Lin–Chu and Fisher-type stationarity tests due to relatively long period of our panel. The results indicate that all the variables are stationary at level except air pollution (PM_2.5_), air pollution squared (PM_2.5Sq_) and education at first difference shown in Table 2.

Table 3 summarizes the description of the variables and summary statistics. We have observed large variation in the climate change variables i.e., air pollution and temperature across countries over the years. The mean air pollution (PM_2.5_) is about 49.7844 µg/m^3^ with a standard deviation of 25.1282 µg/m^3^. In addition, the mean annual temperature is about 21.9 ℃ with a standard deviation of 9.4 ℃.

Table 4 demonstrates the results of the fixed-effect model between air pollution and neonatal deaths due to acute respiratory infection. The results verify the positive and significant association between air pollutants (PM_2.5_) and neonates’ deaths among Asian countries with a coefficient value of 0.4390, and 0.3882 having *p*-value < 0.01 respectively. The results reveal that a 10 µg/m^3^ increase in PM_2.5_ brings 0.4390 (95% CI: 0.2–0.7) growth in number of neonatal deaths due to acute respiratory infection in Asian countries. Additionally, a positive and significant relationship was found between temperature and neonates deaths. The coefficient values of temperature (0.0254) and temperature Sq (0.0008) with *p*-value < 0.05 reveal that an increase in 1 ℃ in temperature correspond to 0.0254% (95% CI: 0.01–0.04) growth in the number of neonatal deaths in 12 Asian countries. Our results are in line with other studies available in literature [32,33,34]. The seminal outcome of the present study endorses that the neonates in Asian countries are more vulnerable to air pollution and consequent mortality as compared with temperature. Moreover, the socioeconomic variables, i.e., income and education, correspond with neonatal deaths due to increased income and education warranting better health care facilities, mitigating the mortality rate.

However, the population density has demonstrated positive association with neonatal deaths. The income variable is statistically significant while education and population density variables are not statistically significant at 5% level of significance. The coefficient values of income, education, and population density are −0.6951 (*p*-value < 0.01), −0.0160 (*p*-value > 0.05), and 0.1093 (*p*-value > 0.05), respectively, revealing that a 10-unit increase in income results in a 0.6951% decrease in neonatal deaths because of acute respiratory infection in selected Asian countries. These results are in line with the previous studies available in literature [12,35,36,37,38].

## 4. Discussion

The study employed the panel data approach to test the correlation between air pollution (PM_2.5_) and neonatal deaths triggered by acute respiratory infection in Asian countries. The study found that air pollution (PM_2.5_) has a significant and positive association with neonatal deaths attributable to acute respiratory infection. Our results show that exposure to anthropogenic particulates (PM_2.5_) is harmful to child health and growth. Our findings are in line with previous studies validating a positive association between air pollution and neonatal deaths [33,34]. A survey-based air pollution study conducted by Nashville was the leading study regarding chronic exposure to air pollution and consequent neonatal deaths [39]. Studies by [26] have also attested the impacts of air pollution on infant mortality, especially neonatal mortality in low and middle income countries. Regarding developed countries, similar outcomes were found by [40,41,42].

The rapid increase of the Asia Pacific region’s share in the world gross domestic product from 12.7% to 31% in 2013 has increased energy consumption of this region to 45% of the overall global energy consumption [43]. The increased consumption of energy and usage of inefficient energy technologies in industrial and household has relatively intensified air pollution, especially in the urban areas, and consequent high concentration of particulate matter in cities [44]. The population weighted PM_2.5_ concentration in South Asia has increased by 24% between 1990 and 2015 due to faster economic growth [45]. The increase in air pollution (PM_2.5_) has two-fold implications for neonates. Firstly, studies have attested that pregnant mothers, when exposed to air pollution, entail potential hazards for their neonates like lower birth weight, preterm birth, and lung developmental defects causing onset of respiratory diseases and reduced lung function in children [46,47,48,49,50,51]. The similar premise has been endorsed by mechanistic evidence underlining the diverse impacts of air pollution in neonates’ biotics like affected cellular and molecular system causing altered immune response and abnormal respiratory functions and lung diseases. Secondarily, Neonates aged 0–28 days demonstrate an exceptional spectrum of diseases comprising clinical presentations like “acute life-threatening events” and “respiratory distress syndrome” [52]. Neonates need more air intake for survival and therefore inhale excessive oxygen as compared to children; air polluted with unwanted contaminants enters their lungs and provokes consequent deaths. Neonates breathe excessively through their mouth cavity and bypass the nasal filter, permitting the entry of a large number and variety of pollutants in their lower airways [53]. Therefore, pollutants variant in size and chemical nature either accumulate or pass through the lung tissues affecting the respiratory system made up of different mucosal cilia and protective mechanisms [9]. Normal vapors of pollutants are either absorbed through human tissues or processed as body fluids depending on hydrophilicity and hydrophobicity, but PM_2.5_ invades the lungs and becomes a notable health risk determinant [15,16,17]. Malevolent effects of air pollutants have been seen in different respiratory diseases like respiratory infections [54,55,56]. Chronic obstructive pulmonary disease [57] and asthma [58,59] are the diseases attributable to air pollutants.

The results of the study also show positive association between temperature and neonates deaths caused by acute respiratory infections. Climate change unswervingly threatens respiratory health by aggravating or promoting respiratory diseases or indirectly by increasing exposure of the risk factors for respiratory diseases [60]. Temperature, sunshine duration, relative humidity, and concentration of pollutants were significantly correlated to acute respiratory infections. In the similar perspective, temperature extreme has been reported as a moderating factor for acute respiratory events and exacerbations of existing respiratory diseases causing mortality [61]. Ambient temperature as an abiotic feature has strong association with lungs function and corresponding respiratory health disorders [61].

The authors further validated income and education level negatively affecting the neonates’ deaths. An increased income enhances an individual’s demands with reference to quantity, quality of goods and services, and utilization of public and private health facilities for themselves and their siblings, which improves overall health status [37,38]. An increased income has positive association with the government’s provision of better resources for public health services, thus reducing mortality. Education is also an important determinant for promoting health awareness. Parental education ensures neonates’ improved health due to better access to healthcare and prevention and cautious life patterns of the parents. Well informed parents demonstrate diagnostic and prognostic measures for the better health of the neonates. However, low level of education in developing countries remains a critical factor in reducing the mortality rate of children. Whereas, population density shows a positive relationship with neonates’ deaths in Asian countries, which are densely populated.

## 5. Conclusions

Air pollution is the most fatal environmental threat in emerging Asia affecting masses at a higher rate than those in developed countries. The impact of air pollution (PM_2.5_) on children’s health has received great attention in recent years. Ambient air pollution may cause the onset of acute respiratory infection [15,16,17], premature birth and low birth weight [46,47,48]. This study evaluated the association of air pollution (PM_2.5_) and varying temperature on neonatal deaths due to acute respiratory infection during 2000–2017 in 12 Asian countries. Through the panel fixed-effect model, we observed positive and significant association among air pollution (PM_2.5_), temperature, and neonatal deaths. Our results also provide evidence regarding the associated role of other socio-economic variables like income, education, and population density in neonates’ mortality. 

### Policy Implications

The present study provides useful insights for policy makers and stakeholders to encounter the advent of air pollution and associated problems causing mortality in children. Concrete policies on deterring the usage of fossil fuels for domestic and industrial purposes and ensuring availability of inexpensive and environment-friendly alternate energy sources will better assist in combating air pollution and confounded risk. Cheaper availability of automobiles powered by solar energy or hybrid vehicles can assist in mitigating pollution and associated problems. The government needs to restrict installation of industries in urban areas and proper management of industrial waste to check the outbreak of respiratory diseases and consequent diseases, morbidity, and mortality in neonates. In the health sector, counseling of pregnant mothers by healthcare professionals and communication about pollution and embedded dangers for neonates can also assist in controlling mortalities. Availability of life-saving gadgets like incubators and post-natal management units in mother–child health centers of the selected Asian countries will positively contribute to control of neonate’s death. 

## Figures and Tables

**Table 1:** Number of neonatal deaths due to acute respiratory infection over the study period between 2000 and 2017.

Year	Neonatal Deaths											
	Bangladesh	China	India	Indonesia	Mongolia	Myanmar	Nepal	Pakistan	Philippines	Sri Lanka	Thailand	Viet Nam
2000	12,232	51,491	86,337	6518	66	2890	2501	19,953	2576	186	825	2011
2001	11,576	45,447	82,660	6341	62	2844	2342	19,309	2569	163	753	1889
2002	10,917	39,718	78,860	6170	59	2764	2175	18,731	2542	144	679	1798
2003	10,246	35,674	74,981	6021	57	2666	2006	18,209	2501	129	610	1731
2004	9558	31,101	71,223	5886	55	2548	1848	17,788	2449	122	543	1695
2005	8878	25,992	67,401	5675	55	2423	1705	17,505	2386	112	487	1670
2006	8233	21,277	63,692	5513	55	2284	1571	17,451	2302	111	443	1656
2007	7594	17,079	59,764	5347	54	2144	1441	17,467	2221	106	405	1629
2008	6987	14,566	56,021	5144	54	2039	1316	17,542	2153	97	370	1604
2009	6409	12,748	52,302	4954	53	1901	1194	17,570	2097	87	338	1576
2010	5876	10,960	48,680	4729	51	1805	1085	17,448	2048	79	308	1545
2011	5386	10,052	45,219	4537	49	1723	985	17,271	2000	73	280	1529
2012	4953	8804	42,112	4364	47	1659	902	17,028	1957	67	254	1511
2013	4579	7704	39,121	4195	45	1600	835	16,704	1914	62	230	1491
2014	4252	6578	36,528	4051	42	1542	780	16,442	1872	58	213	1459
2015	3984	5413	34,201	3898	39	1488	737	16,081	1829	55	195	1412
2016	3732	4951	32,843	3741	37	1444	703	15,713	1787	51	182	1355
2017	3576	4213	31,235	3423	32	1354	645	15,212	1543	45	176	1236
2000-17	12,8969	35,3767	100,3181	90508	911	37117	24771	31,3424	38745	1745	7291	28797

Source: World Health Organization (WHO) and Maternal and Child Epidemiology Estimation Group (MCEE) estimates 2018.

**Table 2:** Levin –Lin–Chu and Fisher-type test results.

Variable	At Level		1^st^ Difference
Respiratory Disease	−2.9158 (0.0018)		
Air Pollution			−21.8254 (0.0000)
Air Pollution Squared			−22.3539 (0.0000)
Temperature	−3.8611 (0.0001)		
Temperature Squared	−4.0398 (0.0000)		
Income	−2.2368 (0.0126)		
Education			−23.8936 (0.0000)
Population Density	−1.7110 (0.0435)		

Note: *p*-values are in parenthesis.

**Table 3:** Variable description and descriptive statistics.

Variables	Description	Source	N	Mean	Std. Dev.	Min	Max
Respiratory Disease	Natural Log of the Total Number of Neonatal Deaths due to Acute Respiratory Infection	UNICEF	215	7.6330	2.0411	3.4657	11.3660
Air Pollution	PM_2.5_ Mean Annual Exposure (Micrograms Per Cubic Meter)	WB	215	49.7844	25.1282	11.0996	100.7844
Air Pollution Squared	PM_2.5_ Squared	WB	215	3106.982	2899.357	123.2015	1010157.5
Temperature	Annual Average Temperature	WB	216	21.9201	9.4095	−0.7909	30.9367
Temperature Squared	Temperature Average Squared	WB	216	568.6241	317.859	0.0039	957.0794
Income	Natural Log of GDP per Capita	WB	215	7.3981	0.7275	5.8487	8.8996
Education	Natural Log of secondary School Enrollment (% Gross)	WB	215	4.1715	0.3493	2.9963	4.7927
Population Density	Natural Log of Population Density (people per sq. km of land area)	WB	215	5.0275	1.5103	0.4339	7.1429

**Note:** where UNICEF and WB are United Nations International Children’s Emergency Fund and World Bank respectively.

**Table 4:** Fixed-effect results.

Respiratory Disease	Coefficient Beta	Std. Err.	*t*-Statistic	Prob	[95% Conf. Interval]	
**Air Pollution Variables**						
PM_2.5_	0.4390 ***	0.1120	3.92	0.000	0.2182	0.6598
PM_2.5_Sq	0.3882 ***	0.0970	4.00	0.000	0.1970	0.5794
Temperature	0.0254 ***	0.0069	3.69	0.000	0.0118	0.0389
Temperature Sq	0.0008 **	0.0003	2.55	0.011	0.0002	0.0014
**Socioeconomic Variables**						
Income	−0.6951 ***	0.1065	−6.53	0.000	−0.9050	−0.4851
Education	−0.0160	0.0380	−0.42	0.673	−0.0909	0.0588
Population Density	0.1093	0.0704	1.55	0.122	0.2482	0.0296
Constant	−0.0275 *	0.0157	−1.75	0.082	−0.0585	0.0035
R^2^	0.7016					
Observations	215					
Prob > F	0.0000					

Note: *** *p* < 0.01, ** *p* < 0.05, * *p* < 0.1.
